# Preparation and Characterization of Resorbable Bacterial Cellulose Membranes Treated by Electron Beam Irradiation for Guided Bone Regeneration

**DOI:** 10.3390/ijms18112236

**Published:** 2017-10-25

**Authors:** Sung-Jun An, So-Hyoun Lee, Jung-Bo Huh, Sung In Jeong, Jong-Seok Park, Hui-Jeong Gwon, Eun-Sook Kang, Chang-Mo Jeong, Youn-Mook Lim

**Affiliations:** 1 Advanced Radiation Technology Institute, Korea Atomic Energy Research Institute, 1266 Sinjeong-dong, Jeongeup-si, Jeollabuk-do 56212, Korea; asj@kaeri.re.kr (S.-J.A.); sijeong@kaeri.re.kr (S.I.J.); jspark75@kaeri.re.kr (J.-S.P.); hjgwon@kaeri.re.kr (H.-J.G.); ymlim71@gmail.com (Y.-M.L.); 2 Department of Prosthodontics, Dental Research Institute, Institute of Translational Dental Sciences, BK21 PLUS Project, School of Dentistry, Pusan National University, Yangsan 50612, Korea; romilove7@hanmail.net (S.-H.L.); cmjeong@pusan.ac.kr (C.-M.J.); 3 Department of Prosthodontics, In-Je University Haeundae Paik Hospital, Busan 48108, Korea; prosth-kang@hanmail.net

**Keywords:** bacterial cellulose membrane, guided bone regeneration, electron beam irradiation, resorbable barrier membrane, optimal radiation dose

## Abstract

Bacterial cellulose (BC) is an excellent biomaterial with many medical applications. In this study, resorbable BC membranes were prepared for guided bone regeneration (GBR) using an irradiation technique for applications in the dental field. Electron beam irradiation (EI) increases biodegradation by severing the glucose bonds of BC. BC membranes irradiated at 100 kGy or 300 kGy were used to determine optimal electron beam doses. Electron beam irradiated BC membranes (EI-BCMs) were evaluated by scanning electron microscopy (SEM), attenuated total reflectance-Fourier transform infrared (ATR-FTIR) spectroscopy, thermal gravimetric analysis (TGA), and using wet tensile strength measurements. In addition, in vitro cell studies were conducted in order to confirm the cytocompatibility of EI-BCMs. Cell viabilities of NIH3T3 cells on 100k and 300k EI-BCMs (100 kGy and 300 kGy irradiated BC membranes) were significantly greater than on NI-BCMs after 3 and 7 days (*p* < 0.05). Bone regeneration by EI-BCMs and their biodegradabilities were also evaluated using in vivo rat calvarial defect models for 4 and 8 weeks. Histometric results showed 100k EI-BCMs exhibited significantly larger new bone area (NBA; %) than 300k EI-BCMs at 8 weeks after implantation (*p* < 0.05). Mechanical, chemical, and biological analyses showed EI-BCMs effectively interacted with cells and promoted bone regeneration.

## 1. Introduction

Various techniques have been used to increase the success rate of tissue regeneration in the dental field [1,2,3,4,5,6,7]. In particular, guided bone regeneration (GBR) is a well-known and widely used technique that uses barrier membranes to prevent the infiltration of soft tissue to bone augmented regions [8].The barrier membranes used for GBR should have the characteristics of cell occlusiveness, wound stabilization, space-making, and provide a stable environment for bone regeneration [9]. Barrier membranes are classified as resorbable or non-resorbable [10]. Non-resorbable membrane materials include polytetrafluoroethylene (PTFE) and titanium mesh, whereas, resorbable membrane materials include polyglycolic acid (PGA), alginate, polylactic acid (PLA), and collagen [11,12,13,14]. Non-resorbable membranes need an additional surgical removal procedure for their removal to prevent wound dehiscence [15]. However, this additional procedure can cause infection, undesirable bone resorption, and have other undesirable side effects [16,17]. Resorbable membranes offer many other advantages over non-resorbable membranes, for example, they provide better soft tissue healing, are cheaper to produce, and have lower complication risks [18].

Bacterial cellulose (BC) has been produced by in vitro synthesis using the Gram-negative bacterium Gluconacetobacter xylinum [19]. BC membranes (BCMs) are composed of 3-dimensional (3D) nano-fibrous networks of linear polysaccharide polymer linked by β-(1,4) glycosidic linkages [20]. Typically, BCMs have good mechanical properties, high levels of crystallinity, high water holding capacities, interconnected 3D porous nanostructures, and excellent biocompatibilities [21,22,23,24]. These characteristics can be advantageous for regeneration of body organs, such as, skin, bone, cartilage, nerves, heart, and blood vessels [25,26,27]. Recently, many studies have examined potential uses for BCM in the dental field [28,29]. The structure of BCMs is similar to that of collagen membrane, which is the material most widely used for resorbable barrier membranes for GBR [30]. 

However, there is an important limitation to the use of BCM to replace the collagen membrane. BCM is not biodegradable in the human body because of a lack of cellulose degrading enzymes (cellulases) [31]. To overcome this problem, various methods, such as, acid hydrolysis, alkaline hydrolysis, delignification by oxidation organosolv pretreatment and pretreatment with ionic liquids, have been proposed to accelerate the hydrolysis of cellulose [32,33,34,35,36]. However, these methods have disadvantages, such as, difficulty accurately controlling degradation and potential cytotoxicity due to residual chemicals in BCMs for clinical application [31,33,36]. 

Therefore, in the present study, an electron beam irradiation (EI) processes was used to control the biodegradability of BCMs. Several radiation-based techniques based on gamma ray, electron beam, or ion beam irradiation have been used to crosslink, graft, or degrade polymers and thus modify their properties [37]. In particular, it has been reported that high energy EI effectively reduces natural polymer molecular weight and mechanical properties without the need for additional chemicals [38,39,40,41]. The purpose of this study was to prepare BCMs irradiated with different electron beam doses and to determine their mechanical, chemical, and biological properties. In addition, an in vivo study was conducted using a rat calvarial defect model to optimize the electron beam irradiation process in terms of bone regeneration and biodegradability.

## 2. Results and Discussion

### 2.1. Characterization of EI-BCMs

#### 2.1.1. Scanning Electron Microscopy

**Figure 1:**
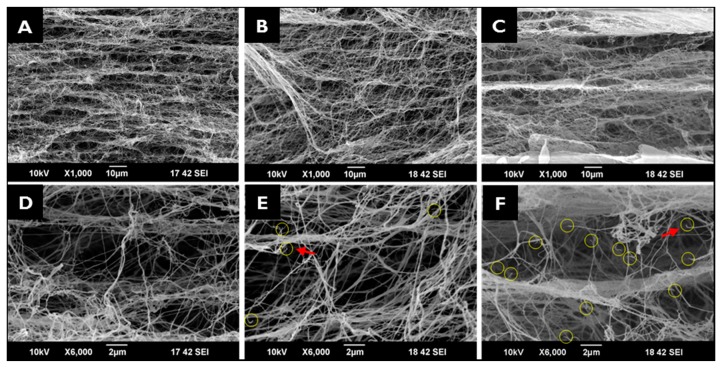
Cross-sectional SEM images: (**A**,**D**) NI-BCM; (**B**,**E**) 100k EI-BCM; (**C**,**F**) 300k EI-BCM. Yellow circles and red arrows indicate cleaved BC nanofibers in 100k (**E**) and 300k EI-BCMs (**F**).

Figure 1 presents SEM images of NI-BCMs, and 100k and 300k EI-BCMs. All BCMs had a porous multilayered structure of entangled nanofibers and cross-linked by nanofibers between layers. Cleaved BC nanofibers were observed in 100k and 300k EI-BCMs, but 3D porous structures were not affected. BC nanofiber cleavages are considered to result from D-glucose chain and hydrogen bond cleavage [42]. 

#### 2.1.2. Mechanical Properties Analyses

**Figure 2:**
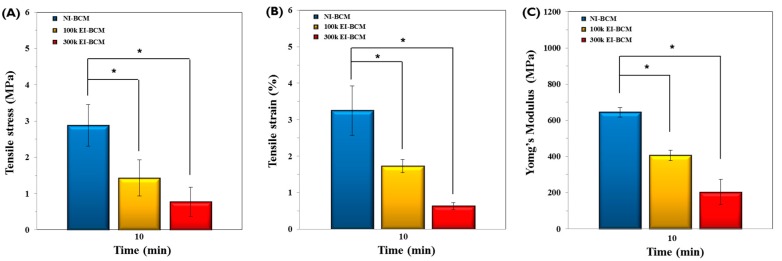
Mechanical properties of BCMs with respect to radiation dose rate after a 10-min soak in water. (**A**) Tensile stress (MPa), (**B**) Tensile strain (%), and (**C**) Young’s modulus (MPa). The mechanical properties of 100k and 300k EI-BCMs were significantly lower than those of NI-BCMs (* *p* < 0.05).

As depicted in Figure 2, mechanical properties, such as wet tensile stress, wet tensile strain, and Young’s modulus of 100k and 300k EI-BCMs were significantly lower than those of NI-BCMs (*p* < 0.05), and those of 300k BCM were the lowest, which were attributed to cleavages observed by SEM [42]. It has been previously reported that chain cleavage of BC nanofibers resulted in significant reductions in mechanical properties [38,39,40,41], and that resorbable membranes used for GBR procedures should have sufficient mechanical strength to attach tightly to bone defects, to prevent sagging and avoid rupture during surgery [43,44]. Although the mechanical properties of EI-BCMs were reduced by irradiation, the mechanical properties of 100k EI-BCMs were similar to those of collagen membranes, as we previously reported [30].

#### 2.1.3. Attenuated Total Reflection-Fourier Transform Infrared Spectroscopy (ATR-FTIR) 

**Figure 3:**
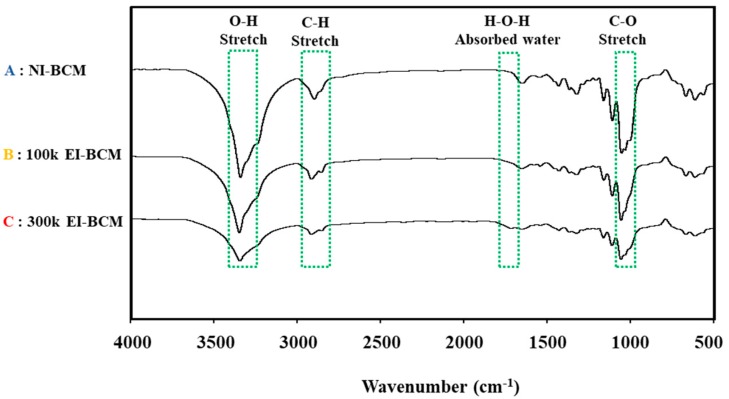
ATR-FTIR spectra of **A**: NI-BCM, **B**: 100k EI-BCM and **C**: 300k EI-BCM. The results obtained indicated that cleavage occurred in the main chain of the BC through change of chemical characteristics.

ATR-FTIR was performed in order to determine the effect of irradiation dose on molecular changes in BCMs. The IR spectra of all three BCMs had peaks at 3410, 2900, 1642, and 1060 cm^−1^. Figure 3 illustrates that the intensity of the O–H group at 3410 cm^−1^, of C–H stretch at 2900 cm^−1^, of H–O–H bending of absorbed water at 1642 cm^−1^, and of the C–O–C pyranose ring skeletal vibration at 1060 cm^−1^ decreased on increasing the irradiation dose. This indicates that the cleavage occurred in the main chain of the BC through change of chemical characteristics [45,46]. It has been previously reported that irradiation causes the degradations of polysaccharides and natural polymers [31,47].

#### 2.1.4. Thermogravimetric Analyses (TGAs)

**Figure 4:**
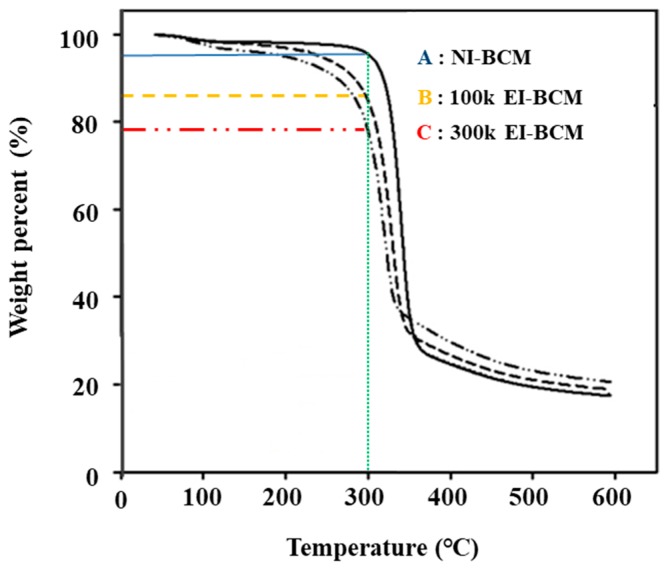
Thermal gravimetric analysis (TGA). NI-BCM had a high decomposition temperature near 300 °C. As radiation dose increased, weight loss rates increased (**A**: NI-BCM, 7%; **B**: 100k EI-BCM, 15%; and **C**: 300k EI-BCM, 22%).

Thermal gravimetric analysis was used to determine the thermal properties of NI-BCM, 100k BCM and 300k BCM. Figure 4 shows the temperature decomposition profiles of these BCMs. NI-BCMs had a high decomposition temperature of near 300 °C. This was attributed to strong inter-chain hydrogen bonds in the crystalline regions of BCM [48]. As the radiation dose was increased, weight loss rates increased (NI-BCM, 7%; 100k EI-BCM, 15%; and 300k EI-BCM, 22%).

#### 2.1.5. In Vitro Degradation

**Figure 5:**
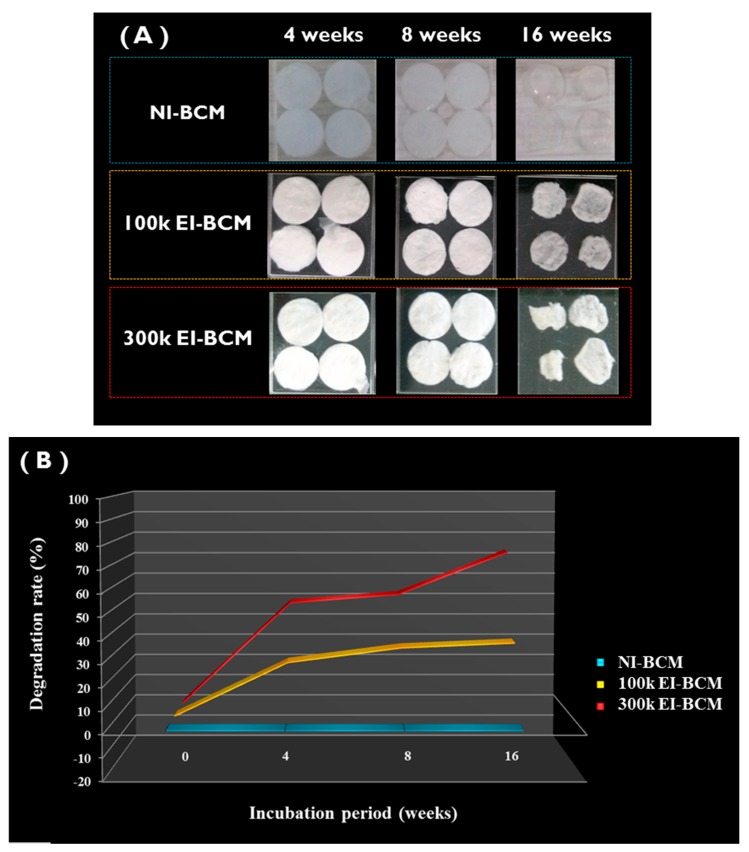
In vitro degradation of BCMs. (**A**) The degradation rates of NI-BCMs, and 100k and 300k EI-BCMs were measured after immersion in PBS for 4, 8, or 16 weeks. (**B**) Degradation rates of EI-BCMs increased with radiation dose, whereas the degradation rate of NI-BCM did not change. 300k EI-BCMs exhibited a weight loss of approximately 70% after 16 weeks.

Figure 5 shows the effect of electron beam dose on BCM degradation in PBS. The degradation rates of NI-BCMs and of 100k and 300k EI-BCMs were measured at 4, 8, and 16 weeks. The degradation rate of EI-BCMs was observed to increase with the radiation dose, whereas the degradation rate of NI-BCMs did not change. In particular, 300k EI-BCMs exhibited a weight reduction of ~70% after 16 weeks in PBS. It has been reported mechanical and chemical changes in biopolymers caused by radiation promote biodegradation [42,32]. This result confirmed that the hydrolysis of the BC polysaccharide chain by EI can be effective for reduction of the molecular weight of BC and accelerated the degradability of the BCMs [39,49,50].

### 2.2. In Vitro Cell Studies

#### 2.2.1. Cell Proliferation Assay

**Figure 6:**
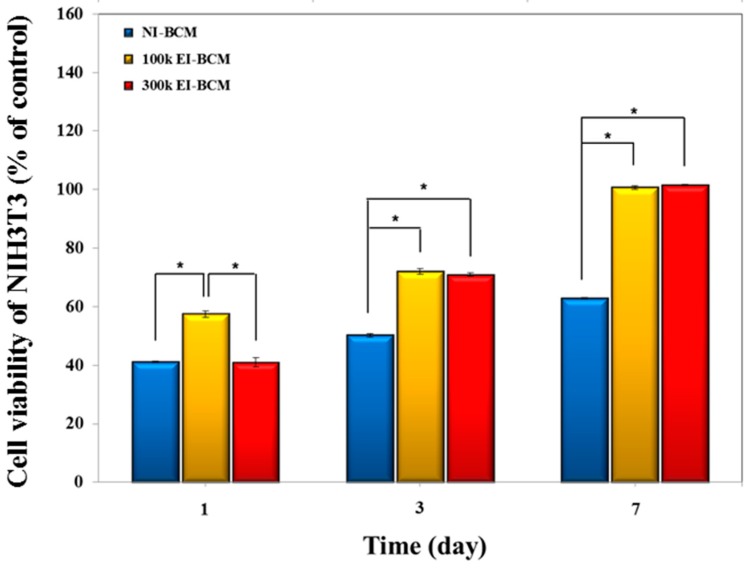
Cell viabilities of NIH3T3 cells cultured on BCMs. CCK-8 assays showed that the viabilities of cells on 100k EI-BCMs and 300k BCMs were significantly greater than those on NI-BCMs after 3 and 7 days (* *p* < 0.05).

CCk-8 assays of cells on the NI-BCMs and 100k and 300k EI-BCMs were conducted in order to determine cell viabilities, adhesions, and proliferations. As depicted in Figure 6, cell viabilities of NIH3T3 cells on 100k and 300k EI-BCMs were significantly greater than on NI-BCMs after 3 and 7 days (*p* < 0.05). After one day, although the initial cell proliferation of NIH3T3 cells on all samples were poorer, cells on 100k EI-BCM proliferated slightly better than cells on NI-BCM. Furthermore, these results demonstrate that the cell viabilities, adhesions, and proliferations are bioactive after the electron beam irradiation process. Because the surface biochemical characteristics of barrier membranes used for GBR influence cell adhesion, the effects of surface modification of BCMs using radiation [51], plasma [52], small signaling peptides [53], and of amino acid (e.g., Arg-Gly-Asp (RGD)) [54] have been investigated with the aim of improving interactions between cells and BC. These modifications of BCM surfaces can change the density in neutral polysaccharides of NI-BCM surface [55]. In the present study, the hydrophilic surfaces of EI-BCMs modified by electron beam irradiation were found to be more bioactive and to promote cell adhesion, viability and proliferation on BCMs.

#### 2.2.2. Immunofluorescent Staining and FE-SEM Analyses of Cells on BCMs

**Figure 7:**
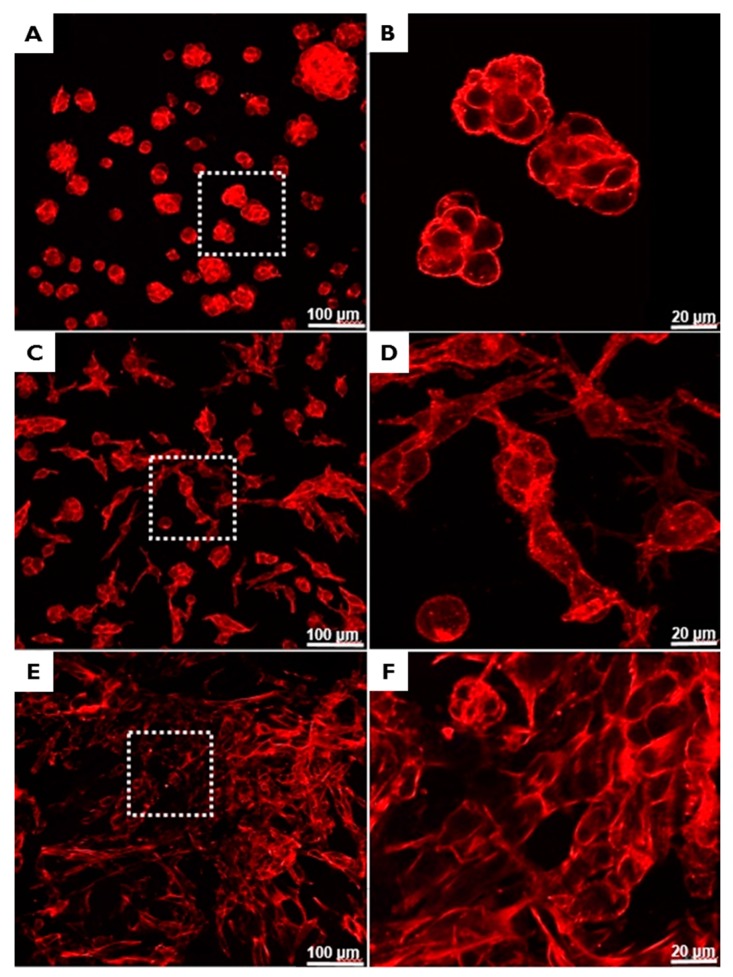
Immunofluorescent staining images obtained by confocal microscopy of adherent cells on NI-BCMs (**A**,**B**); 100k EI-BCMs (**C**,**D**); and 300k EI-BCMs (**E**,**F**). The adherent cells on EI-BCMs were more differentiated and possessed long, straight f-actin stress fibers whereas those on NI-BCMs were circular. Furthermore, degree of cell differentiation increased with irradiation dose.

**Figure 8:**
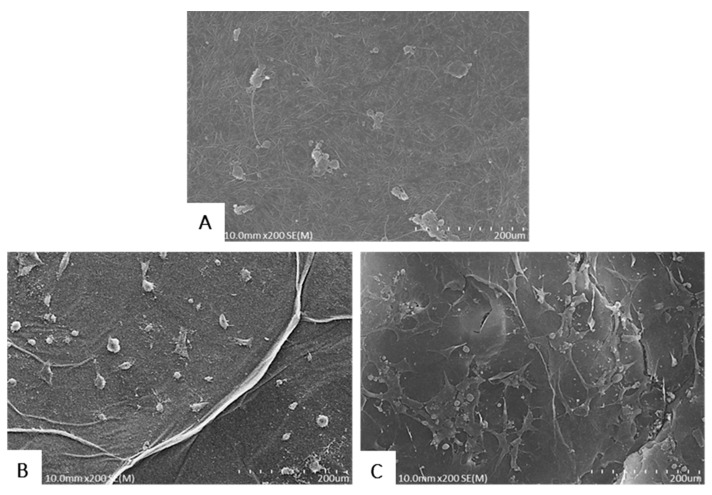
FE-SEM images of adherent cells on NI-BCMs (**A**), 100k EI-BCMs (**B**) and 300k EI-BCMs (**C**). Cells on NI-BCMs were localized, whereas the cell growth on EI-BCMs was induced by the nano-fibrous structure of BC and spread in random directions.

The abilities of BCMs to support NIH3T3 cell adhesion were evaluated by F-actin staining (Figure 7) and FE-SEM (Figure 8). Cells adherent on EI-BCMs were more differentiated and had noticeable long, straight f-actin stress fibers than cells on NI-BCMs, which were circular. While the cells on NI-BCMs were mainly localized, the development of cell growth on EI-BCMs appeared to be guided by their nano-fibrous structures. In addition, cells on 300k EI-BCMs were more differentiated than cells on 100k EI-BCMs.

### 2.3. In Vivo Animal Studies

#### 2.3.1. Histologic Findings

During the initial healing period, mild signs of inflammation, such as, exudate and edema, were observed around grafted BCMs in some rats, but no sign of foreign body or microscopic inflammation was observed. These tissue responses of grafted BC materials have been reported in previous studies [24,56,57,58]. Interestingly, the infection rate of BC in man is so low it is used to produce dressings for wounds and burns [26,55,59]. 

**Figure 9:**
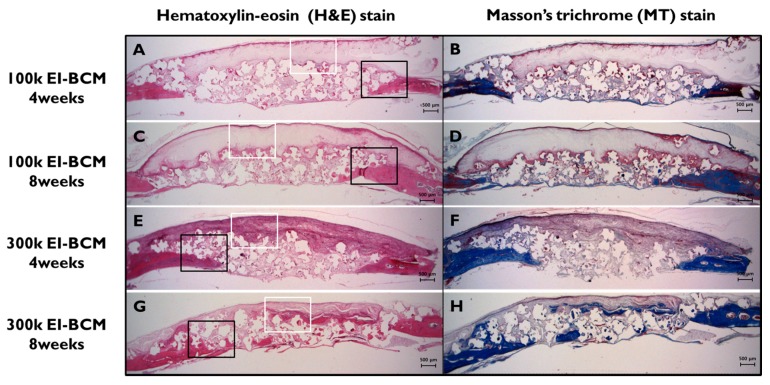
Histological views of defect sites in the 100k and 300k EI-BCM groups. New bone formation and fibrous connective tissue were observed at 4 weeks (**A**,**B**,**E**,**F**) and at 8 weeks after surgery (**C**,**D**,**G**,**H**), and were mainly observed around membranes and old bone. The black rectangles indicate the new bone area in Figure 10 and the white boxes represent the EI-BCMs in Figure 11 (original magnification: 12.5×; (**A**,**C**,**E**,**G**) H&E stained; (**B**,**D**,**F**,**H**) M&T stained).

**Figure 10:**
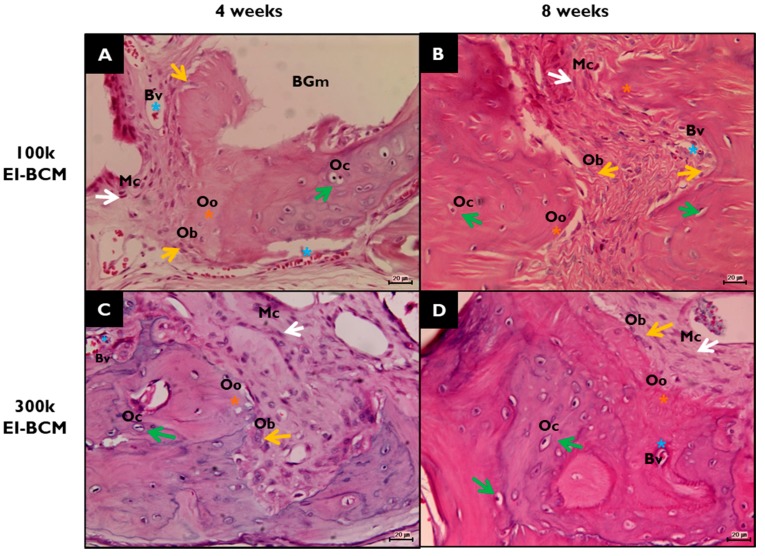
Histological view of new bone areas (**NBAs**) in H&E stained defect sites. (**A**) 100k EI-BCM group at 4 weeks after surgery; (**B**) 100k EI-BCM group at 8 weeks; (**C**) 300k EI-BCM group at 4 weeks; (**D**) 300k EI-BCM group at 8 weeks; **NB**, new bone; **BGm**, remaining graft materials; white arrow, mesenchymal cell (**Mc**); blue asterisk, blood vessel (**Bv**); yellow asterisk, osteoid (**Oo**); yellow arrow, osteoblast (**Ob**); green arrow, osteocyte (**Oc**) within lacuna (Original magnifications: 100×).

**Figure 11:**
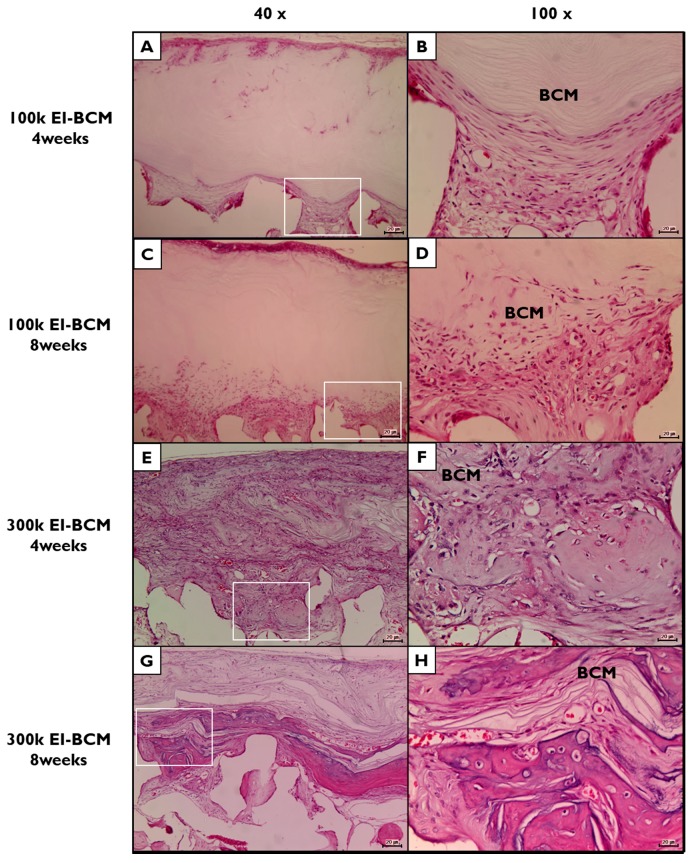
Histological view of electron beam irradiated bacterial cellulose membranes (EI-BCMs) in H&E stained defect sites. (**A**,**B**) 100k EI-BCM group at 4 weeks after surgery; (**C**,**D**) 100k EI-BCM group at 8 weeks; (**E**,**F**) 300k EI-BCM group at 4 weeks; (**G**,**H**) 300k EI-BCM group at 8 weeks; **BCM**, bacterial cellulose membrane. (Original magnifications: (**A**,**C**,**E**,**G**) 40×; (**B**,**D**,**F**,**H**) 100×).

During the 8-week healing period, grafted EI-BCMs did not induce inflammatory responses and integrated with surrounding tissues (Figure 9). Both 100k and 300k EI-BCMs maintained adequate space for bone regeneration and these spaces under membranes were filled with fibrous connective tissue and bone-like materials (Figure 10). No EI-BCM was completely degraded after 8 weeks, but the degradation of 300k EI-BCMs was greater than that of 100k EI-BCMs at 4 and 8 weeks (Figure 11). 

Although the EI process did not change the thickness or nanoporous structure of BCMs [41,42,49] and both EI-BCM specimens for present study were fabricated with similar thicknesses, the more hydrophilic surfaces of the 300k EI-BCM caused more active tissue reactions, leading to more degradation and the remaining membrane was thinner than that of 100k EI-BCM [39,42,47,49,50]. 

#### 2.3.2. Histometric Analyses

**Figure 12:**
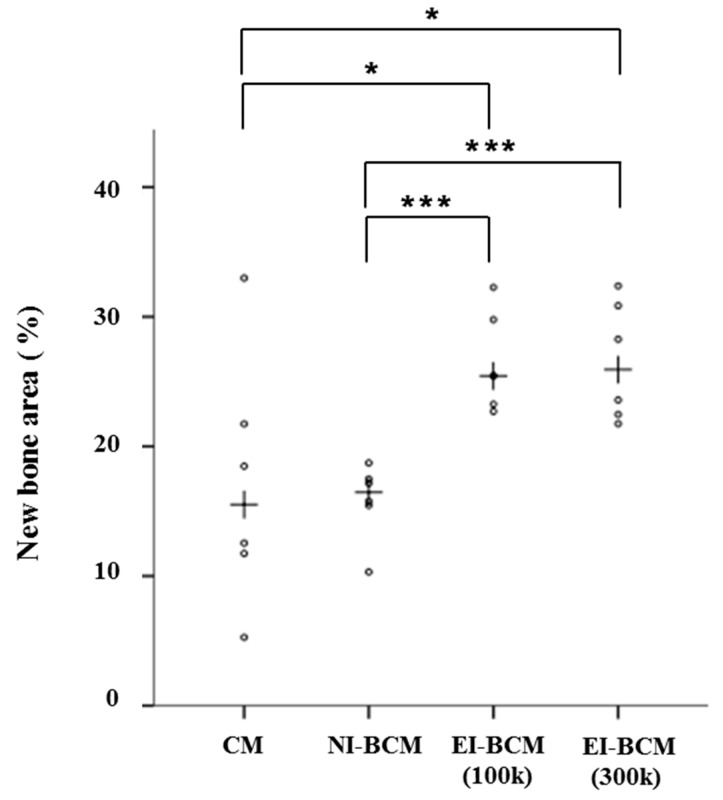
Scatter plots and median (crosses) new bone area percentages (%) at 4 weeks after surgery (* *p* < 0.05, *** *p* < 0.001).

**Figure 13:**
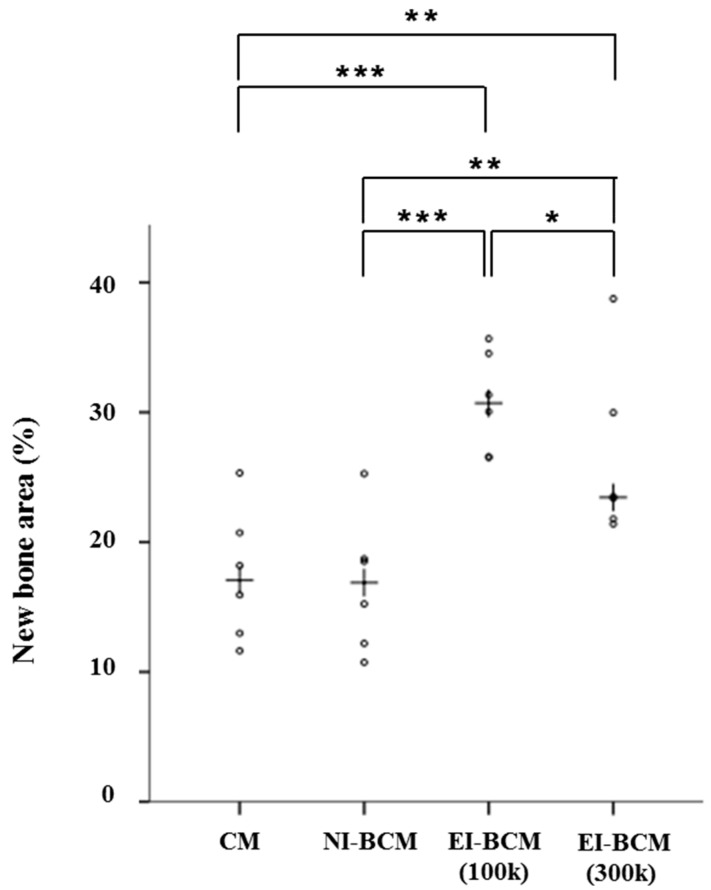
Scatter plots and median (crosses) new bone area percentages (%) at 8 weeks after surgery (* *p* < 0.05, ** *p* < 0.01, *** *p* < 0.001).

Results regarding comparisons of the collagen membrane (CM) and NI-BCM group include results of our previous study [30] (Table 1). The present study was carried out under identical conditions. At 4 weeks, the 100k and 300k EI-BCM groups showed significantly greater new bone areas (NBA; %) than the CM (*p* < 0.001) or NI-BCM groups (*p* < 0.05), but no significant difference was observed between the two EI-BCMs (Figure 12). At 8 weeks, the NBA (%) of 100k EI-BCM group was significantly greater than in the CM group (*p* < 0.001), the NI-BCM group (*p* < 0.001), and in the 300k EI-BCM group (*p* < 0.05) (Figure 13). Based on this analysis, EI-BCM was found to be more effective at promoting bone regeneration than CM or NI-BCM, and 100 kGy was better than 300 kGy for producing resorbable BCMs.

## 3. Materials and Methods

### 3.1. Preparation of Bacterial Cellulose Membranes (BCMs)

BCMs (Jadam Co., Jeju, Korea) were produced using the bacterial strain *Gluconacetobacter hansenii TL-2C*, which was incubated for 7 days in a static culture containing 0.3% (*w*/*w*) citrus fermented solution and 5% (*w*/*w*) sucrose at pH 4.5 (adjusted using acetic acid). The obtained gel-like pellicles of BC were purified by immersion in deionized water at 90 °C for 2 h, and then boiled in 0.5 M NaOH for 15 min in order to remove bacterial cell remnants. The BC obtained was washed several times with deionized water and soaked in 1% NaOH for 2 days. Finally, the alkali was removed from the pellicles by washing. All other reagents and solvents were of analytical grade and used without further purification.

### 3.2. Fabrication of Electron Beam Irradiated BCMs (EI-BCMs) 

**Figure 14:**
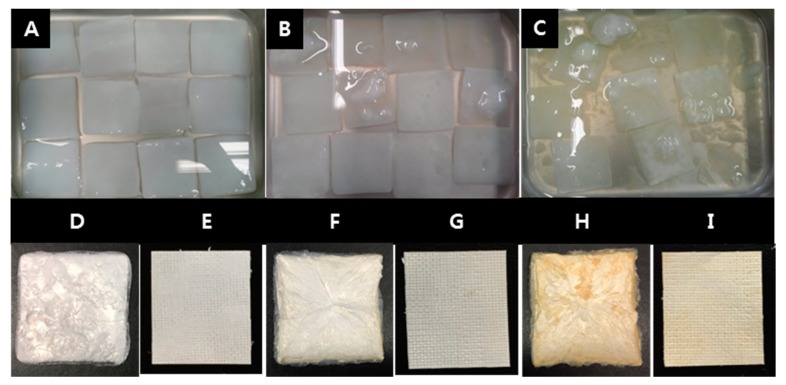
Bacterial cellulose (BC) pellicles were irradiated in distilled water by Electron beam linear accelerator (**A**: NI-BCMs, **B**: 100k EI-BCMs, **C**: 300k EI-BCMs). After the radiation, the BC pellicles were lyophilized (**D**: NI-BCMs, **F**: 100k EI-BCMs, **H**: 300k EI-BCMs) and then compressed into sheet (**E**: NI-BCMs, **G**: 100k EI-BCMs, **I**: 300k EI-BCMs).

Initially, BC pellicles washed with distilled water were irradiated at room temperature using an electron beam linear accelerator (10 MeV, 0.5 mA) at the Korea Atomic Energy Research Institute (Jeongup, Korea) at a dose rate of 5 kGy/min to doses ranging from 100 kGy to 300 kGy. Pellicles were the washed with deionized water, fixed between stainless steel wire meshes to remove water and then compressed for 5 min into sheets using a press (Carver 3969, Wabash, IN, USA) and dried in a freeze dryer at −80 °C for 48 h. Finally, the 100k and 300k EI-BCMs (100 kGy and 300 kGy irradiated BC membrane) and non-irradiated BCMs (NI-BCMs; controls) were prepared (Figure 14).

### 3.3. Characterization of EI-BCMs

#### 3.3.1. Scanning Electron Microscope (SEM) Image Analysis of BCMs

SEM images of NI-BCMs, 100k and 300k EI-BCMs were obtained using a JSM-6390 unit (JEOL, Tokyo, Japan) at 10 kV and distance of 10–12 mm. Samples were placed on steel plates and coated with gold for 60 s.

#### 3.3.2. Mechanical Properties 

The mechanical properties of NI-BCMs and 100k and 300k EI-BCMs were determined using a Universal Testing Instrument (Instron 5569, Instron Corp., Canton, OH, USA) equipped with a 5 kN load cell at a crosshead speed of 10 mm/min. Samples were cut into 2 mm × 15 mm pieces. ASTM standard method D 882-88 was used to determine wet tensile strengths after soaking samples in water for 10 min.

#### 3.3.3. Attenuated Total Reflection-Fourier Transform Infrared Spectroscopy (ATR-FTIR)

NI-BCM and 100k and 300k EI-BCMs also subjected to FTIR spectrophotometry using a Bruker Temsor 37 unit (Bruker AXS Inc., Ettlingen, Germany) over the range 500–4000 cm^−1^ at a resolution of 4 cm^−1^ using >32 scans. Specimens were examined in triplicate to ensure reproducibility.

#### 3.3.4. Thermogravimetric Analysis (TGA)

Thermogravimetric analysis of NI-BCMs and EI-BCMs was performed using a thermal gravimeter (TA Q600, TA Instruments, New Castle, DE, USA). All specimens were dried at 45 °C for 12 h prior to conducting the tests. Specimens (15.9 mg) were placed in a platinum pan and heated at 10 °C/min from 40 °C to 800 °C under a nitrogen flow.

#### 3.3.5. In Vitro Degradation of EI-BCMs

The in vitro degradations of NI-BCMs and 100k and 300k EI-BCMs was undertaken by immersing samples in phosphate buffered saline (PBS) solution at pH 7.4 and simulated body fluid (SBF) at 37 °C. These pre-wetted, irradiated BC membranes were then placed in a 20 mL weighing bottle containing 15 mL PBS and SBF solution. Samples were removed, rinsed with deionized water and freeze dried [60]. Samples were cut into 10 mm diameter circles and immersed in PBS and SBF at 37 °C for 4, 8, or 16 weeks, when they were rinsed, freeze dried, and weighed. The averages and standard deviations were recorded, and rates of weight loss were calculated. The compositions of 1× PBS and 5× SBF are provided in Table 2 [61]. 

### 3.4. In Vitro Cell Studies

NIH3T3 cells (ATCC^®^ CRL-1658^TM^) were cultured in Dulbecco’s Modified Eagle Medium containing 4.5 g/L glucose (DMEM-HG, Gibco BRL, Grand Island, NY, USA) and supplemented with 10% fetal bovine serum and 1% penicillin/streptomycin in a 5% CO_2_ incubator at 37 °C and RH 95%. The medium was changed every two days. 

#### 3.4.1. Cell Proliferation Assay

Cell proliferation was measured using a Cell Counting Kit-8 assay (CCK-8, Dojindo Laboratories, Kumamoto, Japan). Briefly, NIH3T3 cells were seeded at a density of 1 × 10^5^ cells/well on NI-BCMs and 100k and 300k EI-BCMs, and cultured for 1, 3, and 7 days then normalized to the day 1 value to calculate the growth percentage of cells cultured in control media. After incubation, culture media were exchanged with culture medium containing 10% CCK-8 solution. Then, while maintaining the same conditions for 90 min, absorbances were measured at 450 nm using a UV-Vis spectrophotometer (MQX 200 model, Bio-Tek Instruments, Winooski, VT, USA). All experiments were performed in triplicate.

#### 3.4.2. Immunofluorescent Staining

Cells were stained in order to evaluate their morphologies on BCMs. After 24 h of cell culture, BCM samples were fixed using 3.7% MeOH-free formaldehyde in PBS for 10 min at 37 °C, washed in PBS, and permeabilized in cytoskeleton (CSK) buffer (10.3 g sucrose, 0.292 g NaCl, 0.06 g MgCl_2_, 0.476 g HEPES buffer, and 0.5 mL Triton X-100 in 100 mL water, pH 7.2) for 10 min at 4 °C. The cell was then blocked using blocking buffer (1% BSA in PBS) for 1 h at 37 °C, and samples were incubated with rhodamine phalloidin (1:100) and Hoechst 33258 (1:1000; a nuclear stain) (Molecular Probes, Eugene, OR, USA) for 1 h at 37 °C. After washing in PBS, samples were mounted on glass slides, and fluorescent images of stained cells on BCMs were acquired using a Laser Scanning Confocal Microscope (LSM 510, Zeiss, Jena, Germany). Cell areas were obtained from acquired images using Imagepro Plus 4.5 (Media Cybernetics, Silver Springs, MD, USA).

#### 3.4.3. Field Emission-Scanning Electron Microscopy(FE-SEM) of Surface Cells

Samples of NI-BCMs and 100k and 300k EI-BCMs were punched out and sterilized with 70% EtOH. NIH3T3 cells were then seeded at a concentration of 1 × 10^5^ cells/well on sample surfaces. After 24 h of cell culture, samples were washed three times with PBS, and cells were fixed in PBS containing 4% paraformaldehyde for 30 min at room temperature. Cells were then rinsed with PBS for 5 min and dehydrated using an ethanol gradient (50%, 70%, 80%, 95%, and 100% EtOH) for 10 min per step. Samples were dried using a hexamethyldisilazane (HMDS) chemical drying series (3:1, 1:1, and 1:3 EtOH:HMDS followed by 100% HMDS at 15 min each and allowed to air dry). Finally, samples were examined under a field emission-scanning electron microscope (S-4800, Hitachi, Tokyo, Japan).

### 3.5. In Vivo Animal Studies

#### 3.5.1. Experimental Animals

Twenty-four Sprague-Dawley rats (males; weight 250–300 g) were chosen. Animals were housed individually in plastic cages under standard laboratory conditions and had ad libitum access to water and standard laboratory pellets. Animal selection, care, and management, and the surgical protocol and preparation for surgery were conducted in accordance with the guidelines issued by the Ethics Committee on Animal Experimentation at the Korea Atomic Energy Research Institute (KAERI-IACUC-2013-004).

#### 3.5.2. Surgical Procedures

**Figure 15:**
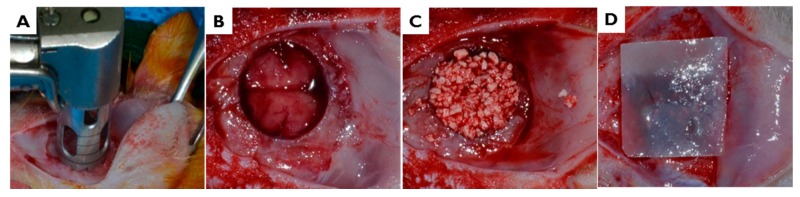
In vivo surgical procedure used to produce rat calvarial defects. (**A**,**B**) In the middle of the cranium, an 8 mm-diameter defect was created with a trephine bur. (**C**) The defect site was treated with HA/*β*-TCP bone graft material, and then (**D**) covered with 100 or 300 kGy irradiated BCM.

After intramuscularly injecting a mixture of xylazine (Rumpun, Bayer Korea, Seoul, Korea) and tiletamine-zolazepam (Zoletil, Vibac Laboratories, Carros, France), surgery was performed under general anesthesia. In each case, the shaved cranial surgical site was disinfected with betadine, and 2% lidocaine HCL (Yu-Han Co., Gunpo, South Korea) was administered for local anesthesia. After making a U-shaped incision, the full-thickness of flap of skin and periosteum was removed. In the middle of the cranium, a standardized 8 mm circular transosseous defect was created with a trephine bur (3i Implant Innovation, Palm Beach Garden, FL, USA). During drilling, the surgical site was washed with saline. After removing the trephinated bony disk, the experimental and control materials were applied. Twelve animals were allocated to each study group. After applying 0.12 mg hydroxyapatite (HA)/β-tricalcium phosphate (TCP) bone graft material (Bio-C, Cowellmedi Implant, Seoul, Korea), the defect site was covered with a 10 × 10 mm membrane of 100k or 300k EI-BCM. The surgical site was closed with 4-0 absorbable sutures (Vicryl^®^, Ethicon, Somerville, NJ, USA) (Figure 15). 

#### 3.5.3. Post-Operative Care and Sacrifice

After surgery, animals received 1 mg/kg gentamicin (Kookje Co., Seoul, Korea) and 0.5 mL/kg pyrin (Green Cross Veterinary Products Co., Seoul, Korea) intramuscularly three times daily for 3 days. Animals were individually caged and received food and water ad libitum. Six animals in each group were allocated a healing period of 4 weeks, and the remaining six animals a healing period of 8 weeks. Animals were sacrificed by CO_2_ inhalation. To collect specimens, defect sites were harvested along with surrounding bone and membranes. Harvested specimens were fixed in neutral buffered formalin (Sigma Aldrich Co., St. Louis, MO, USA) for 2 weeks.

#### 3.5.4. Histometric Analysis

Calvarial specimens were decalcified using 14% ethylenediaminetetraacetic acid (EDTA) and rapid acid decalcification reagents, embedded in paraffin, and sectioned at a thickness of 5 µm in the centers of calvarial defects. The two centermost sections in each block were selected and stained with Hematoxylin-eosin and Masson’s trichrome. Prepared histologic slides were observed under a light microscope (BX50, Olympus, Tokyo, Japan), and images were captured using a CCD camera (Spot Insight 2 Mp, Diagnostic Instruments, Inc., Sterling Heights, MI, USA) fitted with an adaptor (U-CMA3, Olympus, Tokyo, Japan). In order to calculate areas of new bone and residual biomaterials in images, computer-assisted histometric measurements were obtained and percentages of new-bone and residual biomaterials in defect areas were calculated using an image analysis program (Image-Pro Plus, Media Cybernetic, and Silver Spring, MD, USA). 

#### 3.5.5. Statistical Analyses

All quantitative results were obtained by analyzing samples in triplicate. In vitro study results are expressed as means ± SDs. Because data were not normally distributed, non-parametric tests were performed. The statistical analysis was performed using SPSS ver. 23.0 (SPSS, Chicago, IL, USA). Results obtained from the in vivo studies were expressed as means, standard deviations, and medians and statistical analysis was performed using R ver. 3.2.5 (The R Foundation, Vienna, Austria). To compare group histometric results, we used the non-parametric analysis devised by Brunner & Langer. The statistical significance was accepted for *p* values of <0.05.

## 4. Conclusions

The optimal radiation dose required to achieve a suitable level of biodegradation of bacterial cellulose membranes (BCMs) by electron beam irradiation (EI) process is important. In the present study, the mechanical, chemical, and biological characterizations of EI-BCMs prepared at different doses were investigated. High energy electron beams applied to BCMs reduced wet tensile strength, but increased in vitro cell responses and in vivo bone regeneration on calvarial defects. Within the limits of these experiments, it is suggested that BCMs irradiated at 100 kGy are more effective than BCMs irradiated at 300 kGy for clinical application as resorbable membrane for GBR. With regard to clinical utility, further studies are needed including a sufficient period of animal study, a more specific optimal radiation dose, and control of membrane thickness and porosity.

## Figures and Tables

**Table 1:** New bone area percentages within areas of interest (*n* = 6%).

Weeks	Group (Membrane)	Mean ± SD	Median	*** *p*
4	CM	17.13 ± 9.65	15.51	<0.001
	NI-BCM	15.82 ± 2.94	16.46	
	100k EI-BCM	26.48 ± 3.78	25.44	
	300k EI-BCM	26.55 ± 4.56	25.92	
8	CM	17.47 ± 5.09	17.07	<0.001
	NI-BCM	16.78 ± 5.27	16.88	
	100k EI-BCM	30.79 ± 3.86	30.70	
	300k EI-BCM	26.47 ± 6.77	23.45	

CM: collagen membrane; NI-BCM: unirradiated bacterial cellulose membrane; 100k EI-BCM: 100 kGy irradiated bacterial cellulose membrane; 300k EI-BCM: 300 kGy irradiated bacterial cellulose membrane. The symbols ‘***’ indicate statistically significant at *p* values of <0.001).

**Table 2:** Amounts of reagents used to create 1× PBS and 5× SBF.

1× PBS (Phosphate Buffered Saline)			5× SBF (Simulated Body Fluid)		
137.0	mM	NaCl	710.0	mM	Na^+^
2.70	mM	KCl	25.0	mM	K^+^
10	mM	Na_2_HPO_4_·H_2_O	12.5	mM	Ca^2+^
2.00	mM	KH_2_PO_4_	7.5	mM	Mg^2+^
1.00	mM	CaCl_2_	21.0	mM	HCO^3−^
0.50	mM	MgCl_2_	740	mM	Cl^−^
			5.0	mM	HPO_4_^2−^
			2.5	mM	SO_4_^2−^

## Abbreviations

BC	Bacterial cellulose
EI	Electron beam
EI-BCM	Electron beam irradiated BC membrane
NI-BCM	Unirradiated BC membrane
GBR	Guided bone regeneration
CM	Collagen membrane
SEM	Scanning electron microscopy
FE-SEM	Field emission-scanning electron microscope
ATR-FTIR	Attenuated total reflection-Fourier transform infrared spectroscopy analyses
TGA	Thermal gravimetric analyses
NBA	New bone area
PTFE	Polytetrafluoroethylene
PGA	Polyglycolic acid
PLA	Polylactic acid
3D	Three dimension
PBS	Phosphate buffered saline
SBF	Simulated body fluid
HA/*β*-TCP	Hydroxyapatite/*β*-tricalcium phosphate
EDTA	Ethylenediaminetetraacetic acid
